# Incidence of Cardiopulmonary Arrests After Implementation of a Rapid Response System During Operation Time Vs Non-Operation Time

**DOI:** 10.1186/2197-425X-3-S1-A527

**Published:** 2015-10-01

**Authors:** YY Choi, J Min, DS Lee, H Min, EY Lee, G Lim, Y Kim, H Kang, I Song, Y-J Cho, YJ Lee

**Affiliations:** Seoul National University Bundang Hospital, Seongnam, Republic of Korea

## Introduction

Rapid response systems (RRSs) are considered an important tool for improving patient safety.

## Objectives

We studied the effect of an RRS on the incidence of cardio-pulmonary arrests (CPAs).

## Methods

We performed a retrospective before-after analysis of the CPAs in a 1,360-bed tertiary care hospital from January, 2009 to October, 2014. We included 176,193 admissions before and 117,822 admissions after implementing the RRS on October, 2012. the operation time of the RRS was from 7 am to 10 pm during weekdays. the primary outcome was CPA incidence, which was expressed as the case per 1,000 admissions.

## Results

The overall CPA incidence was 1.31. Although the number of admission per month and the case-mix index were increased (3915.4 vs. 4712.9, p= < 0.001; 1.0923 vs. 1.1255, p = 0.003, respectively), the CPA incidence was significantly decreased (1.66 vs. 1.25, p = 0.03), and there was no detectable changes on the mortality (1.27 vs. 1.33, p = 0.273). the CPA incidence in the surgical department was significantly reduced (1.13 vs. 0.38, p = 0.014), however, the reduction of the CPA incidence in the medical department was insignificant (3.93 vs. 2.97, p = 0.075). the implementation of the RRS did not reduce the CPA incidence during non-operation time (0.80 vs. 0.73, p = 0.573), but decreased during operation time (0.86 vs. 0.52, p = 0.001). the immediate survival rate (76.9% vs. 67.2%, p = 0.034) during the operation hours of the RRSs was better than during the non-operation hours.

## Conclusions

The implementation of the RRS reduced the CPA incidence, which was originated in the reduction of the CPA during the operation hours.

